# Role of Neuron and Glia in Alzheimer’s Disease and Associated Vascular Dysfunction

**DOI:** 10.3389/fnagi.2021.653334

**Published:** 2021-06-15

**Authors:** Sanghamitra Bandyopadhyay

**Affiliations:** ^1^Developmental Toxicology Laboratory, Systems Toxicology & Health Risk Assessment Group, CSIR-Indian Institute of Toxicology Research (CSIR-IITR), Lucknow, India; ^2^Academy of Scientific and Innovative Research (AcSIR), Ghaziabad, India

**Keywords:** astrocyte, microglia, amyloid beta, neurofibrillary tangles, neurovascular unit, neurodegeneration, cognition

## Abstract

Amyloidogenicity and vascular dysfunction are the key players in the pathogenesis of Alzheimer’s disease (AD), involving dysregulated cellular interactions. An intricate balance between neurons, astrocytes, microglia, oligodendrocytes and vascular cells sustains the normal neuronal circuits. Conversely, cerebrovascular diseases overlap neuropathologically with AD, and glial dyshomeostasis promotes AD-associated neurodegenerative cascade. While pathological hallmarks of AD primarily include amyloid-β (Aβ) plaques and neurofibrillary tangles, microvascular disorders, altered cerebral blood flow (CBF), and blood-brain barrier (BBB) permeability induce neuronal loss and synaptic atrophy. Accordingly, microglia-mediated inflammation and astrogliosis disrupt the homeostasis of the neuro-vascular unit and stimulate infiltration of circulating leukocytes into the brain. Large-scale genetic and epidemiological studies demonstrate a critical role of cellular crosstalk for altered immune response, metabolism, and vasculature in AD. The glia associated genetic risk factors include *APOE*, *TREM2*, *CD33*, *PGRN*, *CR1*, and *NLRP3*, which correlate with the deposition and altered phagocytosis of Aβ. Moreover, aging-dependent downregulation of astrocyte and microglial Aβ-degrading enzymes limits the neurotrophic and neurogenic role of glial cells and inhibits lysosomal degradation and clearance of Aβ. Microglial cells secrete IGF-1, and neurons show a reduced responsiveness to the neurotrophic IGF-1R/IRS-2/PI3K signaling pathway, generating amyloidogenic and vascular dyshomeostasis in AD. Glial signals connect to neural stem cells, and a shift in glial phenotype over the AD trajectory even affects adult neurogenesis and the neurovascular niche. Overall, the current review informs about the interaction of neuronal and glial cell types in AD pathogenesis and its critical association with cerebrovascular dysfunction.

## Introduction

Alzheimer’s disease (AD) is a prevalent form of dementia, clinically manifested by loss in memory and cognitive decline. As reported, around 50 million people suffer from AD or age-associated dementia, which is projected to exceed 152 million globally by 2050 (Peprah and Mccormack, 2019). However, pure AD comprises less than half of the total cases, while most of the patients predominantly show features of cerebrovascular disease and mixed dementia (Barker et al., 2002). The amyloid β (Aβ) plaques and hyperphosphorylated-tau-induced neurofibrillary tangles (NFTs) in the hippocampus and cortical region have long been considered as the only key pathological features of AD (Jacobs et al., 1992; Jellinger and Bancher, 1998). Nonetheless, over the years significant association of AD with vascular dysfunction has been reported, involving brain metabolic derangements, deregulated Aβ clearance, and a subsequent loss in neuronal homeostasis (Cortes-Canteli and Iadecola, 2020; He et al., 2020a).

AD-related cerebrovascular pathology involves reduced episodic memory, without a loss in hippocampal volume. The vascular abnormalities primarily include cerebral micro- and macro-stroke, hemorrhages, lacunar infarcts, hypoxia, small vessel pathologies, embolism, microbleeds, periventricular and deep white matter hyperintensity (WMH), leukoencephalopathy, hypertensive intracerebral hemorrhages, enlarged perivascular spaces, ischemic lesions, and stroke (Pluta, 2004; Ashok et al., 2016; Kuhn and Sharman, 2020). It has been reported that the production and clearance rate of Aβ is 7.6% and 8.3% per hour respectively in humans, and a shift in this equilibrium towards Aβ production results in Aβ peptide and subsequent plaque deposition in the human brain (Bateman et al., 2006).

An immunohistochemical study in the cortical and hippocampal post-mortem samples of AD brain compared to non-demented elderly controls demonstrated intricate interaction between the neuronal and non-neuronal cells, such as astrocytes, microglia, and vascular endothelial cells in AD pathogenesis and an ultimate neurodegeneration (Kirabali et al., 2020). The astrocytes and microglia independently, as well as collectively, stimulate neuroinflammation and promote pro-inflammatory cytokine release and neuronal loss *via* cellular crosstalk in AD (Kaur et al., 2019). White matter damage, axonal loss, demyelination, and reduced differentiation of oligodendrocyte progenitor cells (OPCs) also participate in the AD-associated loss in synaptic plasticity (Butt et al., 2019; Vanzulli et al., 2020), and OPC damage comprises the initial signs of pathology, detected in the hippocampus of 3xTg-AD mice (Vanzulli et al., 2020). Additionally, observations in 3xTg-AD triple transgenic AD mice showed early white matter dystrophy and myelin damage in the hippocampus and entorhinal cortex, prior to Aβ and tau pathology or signs of cognitive decline (Desai et al., 2009). Although, the tau pathology correlates more strongly with neuronal cells, participation of glial activation in tau-induced neurodegeneration and tangle burden has been reported in the Tg4510 (rTg4510) mouse line (model of Tauopathy) overexpressing Chemokine C-C motif ligand (Joly-Amado et al., 2020). Decreased capillary vascular endothelial growth factor (VEGF) and endothelial nitric oxide (NO) synthase in the superior temporal and calcarine cortices also associated with Tau pathology in AD patients (Provias and Jeynes, 2008). Moreover, genes involved in immune modulation, inflammation, and the metabolism and functions of microglia, astrocytes, and oligodendrocytes have been implicated in neuronal loss and AD (Chen et al., 2020).

Vascular changes, in association with reactive glial cells, trigger oxidative stress, and reactive oxygen species (ROS) generation, which cause neurodegeneration (Bou Khalil et al., 2016; Govindpani et al., 2019). Build-up of amyloid on the cerebral vasculature in cerebral amyloid angiopathy (CAA) generates vascular oxidative stress, exacerbating undesired glial reactivity and cerebrovascular dysfunction (Carrano et al., 2011; Han et al., 2015). Additionally, observations in the aged Tg2576 mice (overexpressing human APP695) not only showed the participation of vascular oxidative stress in CAA but also indicated the significant role of nicotinamide adenine dinucleotide phosphate (NADPH) oxidase-mediated vascular oxidative stress in CAA-induced cerebrovascular changes (Han et al., 2015). CAA is a proven key pathological feature of AD, characterized by Aβ deposition in the wall of arteries, arterioles, capillaries, and blood vessels in the central nervous system (CNS; Revesz et al., 2002). Moreover, breakdown of the neurovascular unit (NVU) that comprises pericytes, neurons, and glia, together with blood-brain barrier (BBB) degeneration and reduced cerebral blood flow (CBF), exacerbates AD neuropathology (Zlokovic, 2011; Parkes et al., 2018).

Both amyloid and vascular concepts are well-studied in AD. Specifically, there have been exploratory studies discerning the cumulative involvements of ROS, inflammation, NVU dysfunction, and changes in the brain microvasculature on AD pathogenesis (He et al., 2020a; Ulamek-Koziol et al., 2020). However, a thorough understanding of the neuronal and vascular impairments, together with the participation of brain cells, in AD is yet awaited. The current review highlights the interaction among different cell types of the brain and enumerates the pathways stimulating AD pathogenesis, focusing on mixed dementia that includes AD-associated plaques, tangles, and vascular dementia.

## Current Theories for AD

Several theories govern AD pathology, with the Aβ plaques and NFTs as the primary pathological hallmarks (Masters et al., 2006; Boscolo et al., 2007). Although the “amyloid hypothesis” appears most prominent in AD, NFT generation even seems common for diverse cerebral stress conditions (Khachaturian, 1985; Mirra et al., 1991; Felician and Sandson, 1999). Additionally, the vascular hypothesis emerged as a key theory, considering cardiovascular, cerebrovascular, and old age-related disorders (Kalaria, 1996; de la Torre, 2000b; Kalaria, 2002a). In reality, a synergism of factors augmenting Aβ plaque generation, NFT formation, and vascular abnormalities promotes neurodegeneration, culminating in AD pathology and severe cognitive impairment (de la Torre, 2000a, b; Iadecola, 2003a, b).

### Amyloid and Tau Hypotheses

The amyloid cascade hypothesis is a dominant theory of AD pathology, proposed for the last 30 years. The hypothesis claims an amyloidogenic processing of Amyloid precursor protein (APP) by beta-secretase (BACE) to generate C-terminal fragment, CTFβ, and cleavage of the latter by gamma-secretase to release Aβ (more pathologic Aβ42 and soluble Aβ40; Hardy and Higgins, 1992). The amyloid cascade hypothesis focuses on Aβ deposit in brain parenchyma as a key component of senile plaques, with mutations in APP and presenilin (PSEN1 and PSEN2) genes that induce onset in familial AD (Nicolas et al., 2016). Moreover, AD being multifactorial, sporadic AD (whose cause is still elusive) involves the association of several etiological factors, mechanisms, and pathways in the brain (Armstrong, 2013; Gong et al., 2018). Further, a reduced non-amyloidogenic processing of APP by α-secretase [a disintegrin and metalloproteinase (ADAM)-10 and ADAM-17] has been shown as a key pathway promoting Aβ generation (Bandyopadhyay et al., 2006a, 2007).

Inflammatory pathways impact the onset and progression of AD involving metabolic and cellular alterations that trigger neurodegeneration, primarily in the hippocampus, entorhinal and temporoparietal cortex (Risacher and Saykin, 2013; DeTure and Dickson, 2019). The glial cells (that normally generate trophic factors for physiological requirements) undergo aberrant reactivity, resulting in the overproduction of pro-inflammatory mediators in the etiopathogenesis of AD. The altered inflammatory signaling pathways promote Aβ-peptide deposition and then oligomerization into senile plaques, tau hyperphosphorylation, and an ultimate synaptic and cognitive dysfunction (Zlokovic, 2011; Webers et al., 2020).

Both, oxidative stress and inflammation catalyzed Aβ plaque formation, where the 5′ untranslated region (UTR)-APPmRNA harboring the iron-responsive element (IRE) and interleukin-1 responsive element regulated APP translation (Rogers and Lahiri, 2004). Factors promoting ROS generation, lipid peroxidation, and pro-inflammatory cytokine levels stimulated amyloidogenic APP processing. They also induced an upregulation in the 5′UTR-mediated APP translation and neuronal Aβ through intricate participation of reactive astrocyte and microglia, observed in the hippocampus and frontal cortex of rats showing AD-like pathology (Ashok et al., 2015; Maurya et al., 2016). Hence, through studies on neuronal and astrocyte cell lines and in TgCRND8 mice, it has been claimed that targeting APP mRNA-5′UTR may be a potential strategy in limiting APP translation and attenuating AD pathogenesis (Bandyopadhyay et al., 2006b,c, 2010, 2013; Tucker et al., 2006; Bandyopadhyay and Rogers, 2014). AD is also characterized by the neuronal accumulation of toxic NFTs, formed from microtubule-associated hyperphosphorylated tau proteins as paired helical filaments (PHFs). The PHFs inhibit the normal microtubule assembly and stability, neuronal growth and morphology, and axonal and dendritic transport, inducing synaptic loss and neurodegeneration (Iqbal et al., 2010; Mietelska-Porowska et al., 2014; Alonso et al., 2018). The increased cerebral NFTs and cerebrospinal fluid (CSF) p-tau levels bear a prominent link with the severity of dementia and cognitive loss (Maccioni et al., 2001, 2006; Ghoshal et al., 2002). Hence, drugs targeted to the NFT attenuated the pathological symptoms of AD (Duff et al., 2010).

The toxic deposits of insoluble Aβ in the hippocampus and cortex promote the formation of neuritic senile plaques, and a neuron-Aβ interaction induces tau phosphorylation through tau kinase activation or by modulating phosphorylated state of tau, as seen in the primary septal and hippocampal neurons (Takashima et al., 1998; Zheng et al., 2002). Microtubule-associated tau proteins then undergo truncations and conformational shifts to form filamentous aggregates or NFTs (Binder et al., 2005). The neuritic plaques and aggregated tau induce proximal astroglial and microglial reactivity, neuritic dystrophy, and an increased Aβ plaque and NFT deposition in the hippocampal neurons. These conditions eventually induce neuronal death, degenerated nerve endings, cognitive decline, and dementia (Laurent et al., 2018; van Olst et al., 2020).

Aβ peptides also interact with neurons, inducing altered tau aggregations. A direct interaction of Aβ with the neuronal membranes and receptors [nAchR, N-methyl-D-aspartate (NMDAR) and α-amino-3-hydroxy-5-methyl-4-isoxazolepropionic acid receptor] trigger abnormal calcium influx and oxidative stress, which lead to the activation of glycogen synthase kinase-3β (GSK-3β), and hence increased tau phosphorylation (Wang et al., 2000; Stancu et al., 2014). *In vitro* as well as *ex vivo* studies in neuroblastoma cell lines and synaptosomes, respectively, demonstrated an enhanced Aβ peptide and α7 nAChR interaction, resulting in increased Tau pathology (Stancu et al., 2014). Additionally, cell culture (cultured hippocampal neurons) and animal-based (3xTg-AD) studies suggested the participation of reactive glial cells in acute and chronic inflammation, and further Aβ-mediated Tau pathology (Saez et al., 2006; Kitazawa et al., 2011; Sy et al., 2011).

### Vascular Hypothesis

Aβ plaques and NFTs relate to neurodegeneration, supported by increased expression of these pathological hallmarks in the neuronal cell body and in abnormal neuronal processes adjoining hippocampal and cortical Aβ deposits (Duyckaerts et al., 1998; Lewis et al., 2001; Ribe et al., 2005; Cohen et al., 2013; Koss et al., 2016; Park et al., 2019). Alternatively, the vascular hypothesis claims that cerebrovascular diseases are prevalent comorbidities in AD, which induce cognitive impairment and dementia (Humpel and Marksteiner, 2005). AD involves a marked alteration in the structure and morphology of vascular cells and tissues, ensuing a dysregulated cortical blood flow, vascular fluid dynamics, and vessel integrity. Arterial spin-labeling magnetic resonance imaging (MRI) as well as ischemic vascular dementia demonstrated a loss in CBF in the cortex of AD patients (Schuff et al., 2009). A reduced blood supply in the occipital cortex of B6.PS2APP mouse model of AD also correlated with the findings in the cortex of AD patients (Weidensteiner et al., 2009).

Moreover, arterial spin labeling MRI [measured in AβPPSWE/PS1ΔE9 (APP/PS1) transgenic mice model of AD] revealed that alteration in CBF, mainly at the frontoparietal cortex and thalamus regions rather than the hippocampus and entorhinal cortex, had a significant link with early AD and vulnerability to AD pathology (Guo et al., 2019). Further, *in vivo*, time-lapse, multiphoton microscopy in the APP/PS1 mouse model of AD demonstrated that microvascular lesions regulate the accumulation and clearance of Aβ, indicating a key link between the dynamic morphology of Aβ plaques and vascular dysfunction (Zhang et al., 2020).

Aging factors, exacerbated by cardiovascular disorders and systemic vascular abnormalities, undergo a synergistic interaction, culminating in cerebrovascular damage and severe cognitive decline (Klohs, 2019). Studies in AD patients through single photon emission computed tomography (SPECT), arterial spin-labeling MRI, and [^18^F] fluorodeoxyglucose positron emission tomography (FDG-PET) indicated a key link between AD and altered metabolic parameters in the temporoparietal cortex (Bonte et al., 1986; Burns et al., 1989; Fazekas et al., 1989; Johnson et al., 2005; Alexopoulos et al., 2012). Hypoperfusion and hypometabolism also appeared as critical features in the angular gyrus (Fazekas et al., 1989; Alexopoulos et al., 2012) and posterior precuneus of parietal cortex in AD patients (Binnewijzend et al., 2016).

Vascular pathology starts with cerebral hypoperfusion, which stimulates glial reactivity and an ultimate neuronal cell death and atrophy, with the loss of CNS functions (Wang et al., 2016). Small vessel disease, structural white matter alterations, dysregulated cerebral vasculature, atherosclerosis, BBB dysfunction, and blood vessel rupture upregulate cortical and hippocampal Aβ levels and induce amyloid fibrillogenesis and neuropathological changes in patients at a risk for AD (Pantoni and Garcia, 1997; Kalaria, 2002b; Kovacic and Fuster, 2012). The vascular inflammatory processes also significantly engage in the pathogenesis and generation of AD. The microvascular endothelial cells that release pro-inflammatory cytokines and chemokines induce BBB disintegration, elevate neurotoxic thrombin release, and augment cerebrovascular oxidative stress, triggering neuronal death and cognitive loss in AD (He et al., 2020a; Iannucci et al., 2020).

An increased activity of the angiotensin-converting enzyme (ACE) that converts Angiotensin I (Ang I) to Angiotensin II (Ang II), together with enhanced vasoconstriction, has been reported in AD (Benigni et al., 2010). Ang II also activates NADPH-mediated oxidative stress that functions as a prime risk factor in AD (Ongali et al., 2014; Halliday et al., 2016; Tarafdar and Pula, 2018). Moreover, augmented perivascular ACE-1 levels promote pathological Aβ misfolding and correlate with the severity of AD, as discerned in post-mortem brain tissues from AD patients (from South West Dementia Brain Bank, University of Bristol) showing a frontal cortex Aβ load and signs of CAA (Miners et al., 2008). In terms of metabolic dysfunctions, individuals with obesity, together with type-II diabetes, insulin resistance, prolonged hyperinsulinemia, atherosclerosis, hypercholesterolemia, or hypertension are at a higher risk of developing AD (Polidori et al., 2012; Borshchev et al., 2019; Hayden, 2019; Lee et al., 2020). Ischemic infarcts, hemorrhagic stroke, capillary degeneration and microvascular damage [characterized by focal constriction and thickened vascular basement membrane in the cerebral leptomeningeal vessels and distal intraparenchymal arteries, arterioles, and capillaries of patients (Thal et al., 2002)] reduce CBF, impair functional hyperaemia, and participate in the amyloid pathogenesis (Lai et al., 2015; Tarantini et al., 2017; Hecht et al., 2018; Solis et al., 2020). Here, structural changes in the vascular basement membrane alter endothelial connectivity and adhesion, leading to BBB damage and a subsequent entry of secreted APP and neurotoxic plasma substances into the brain (Kelleher and Soiza, 2013; Govindpani et al., 2019). The ischemic conditions further promote BACE expression and activity, facilitating APP-amyloidogenic processing in the hippocampus and frontal cortex, as observed in rat and mice bilateral common carotid artery occlusion models of CCH (Ashok et al., 2016; Cai et al., 2017).

**Figure 1:**
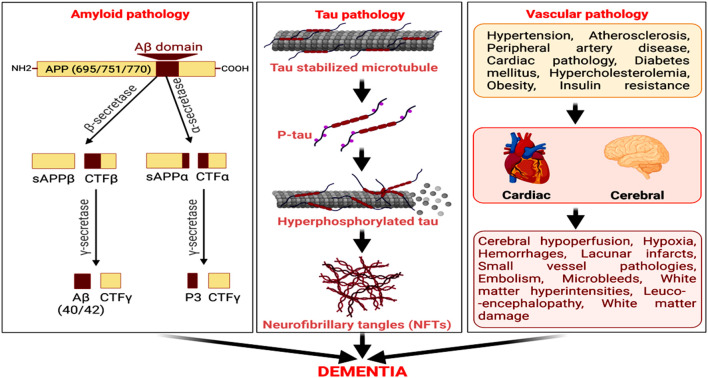
Amyloid, tau, and vascular pathologies as predominant factors for neurodegeneration and cognitive decline in Alzheimer’s disease (AD). The amyloidogenic processing of amyloid precursor protein (APP) by beta-secretase (BACE) and then γ-secretase generates amyloid-β (Aβ). The microtubule-associated protein, tau, undergoes hyperphosphorylation, and self-assemble to form neurofibrillary tangles (NFTs). Cerebrovascular diseases and cardiovascular disorders are also vital risk factors for AD, culminating in dementia.

Cerebrovascular damage and atherosclerotic lesions are also observed in AD patients and have often been considered as predisposing factors to developing the disease (Gupta and Iadecola, 2015; Alosco et al., 2018). White matter hyperintesity and AD-associated neuropathology were detected by MRI in participants from the National Alzheimer’s Coordinating Center’s Data Sets (Alosco et al., 2018). Studies in patients with subcortical stroke, cognitive impairment, and/or dementia or with radiologically defined lacunes revealed that subcortical lacunar infarcts from occluded and perforated arteries associate with reduced cerebral glucose metabolism, forming a contributory factor for the cognitive decline (Kwan et al., 1999; Reed et al., 2001). Hence, cerebrovascular insufficiency and vascular dementia co-exist with enhanced Aβ and senile plaques (Liu et al., 2015; Greenberg et al., 2020). Pathological amyloid accumulation also contributes to the development of subcortical intracerebral hemorrhage and hemorrhagic stroke in elderly AD patients (Vasilevko et al., 2010; Van Nostrand, 2016; Yanagawa et al., 2019). It distinctly connects with clinical conditions, such as intracranial bleeding, stroke, brain ischemia, ruptured aneurysm, head injury, and recurrent seizures. Additionally, diabetes, myocardial infarction, angina, stroke, arterial occlusion, hypertension, and microvascular diseases have strong vascular components and comprise key risk factors for AD (Petrie et al., 2018). Hence, an upregulated amyloidogenic pathway of Aβ generation, tau hyperphosphorylation, NFT formation, vascular damage, and reduced Aβ clearance from the brain are key pathogenic processes underlying AD pathology and related dementia (Figure 1).

**Figure 2:**
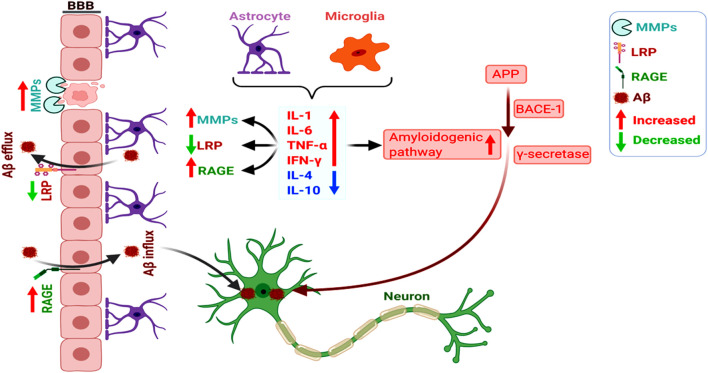
Increased amyloidogenicity, blood-brain barrier (BBB) damage, and dysregulated RAGE and LRP-1 expression induce neuronal Aβ deposition. The pro-inflammatory cytokines (as opposed to anti-inflammatory ones) secreted by the astrocytes and microglia promote the amyloidogenic pathway of APP processing towards Aβ. Glia-mediated inflammation also induces vascular dysfunction involving dysregulated activation of MMPs and altered RAGE and LRP-1-dependent Aβ clearance from the brain, overall increasing neuronal Aβ deposition.

Notably, BBB impairment appears as a key link between amyloid and vascular pathologies. Here, a dysregulated receptor for advanced glycation end product (RAGE; Aβ influx receptor) and low-density lipoprotein receptor-related protein-1 (LRP1; Aβ influx protein) expression and functioning play a central role in impaired vascular clearance of Aβ from the brain, together with the abnormal functioning of glial cells and neurons (Deane et al., 2004; Deane and Zlokovic, 2007). Activated matrix metaloproteinases (MMPs) induce BBB disruption, and the altered Aβ-RAGE and LRP1-interaction disrupt the tight junctions of BBB (Kook et al., 2012). It has also been shown that chronic cerebral hypoperfusion increased MMP9-mediated BBB damage and promoted the hippocampal amyloidogenic APP-processing towards Aβ (Ashok et al., 2016). Overall, the overlap between amyloid production and vascular aberrations accelerates neurodegeneration and AD pathogenesis, with the intricate participation of glial cells, inflammation, and altered cerebral microvasculature (Figure 2; Wanleenuwat et al., 2019).

## Amyloid Hypothesis and Role of Neurons and Glia

### Neuroinflammation

**Figure 3:**
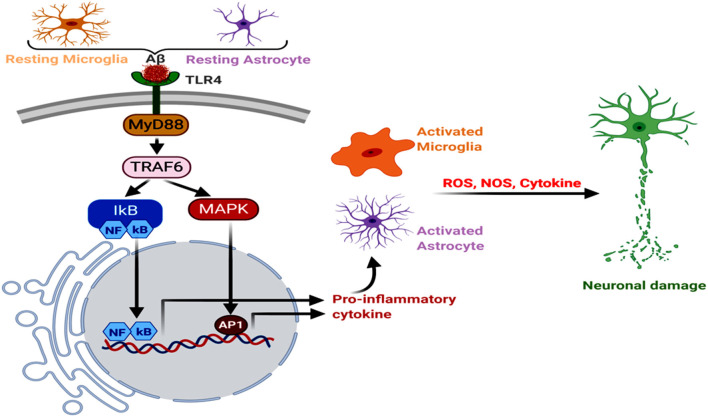
Neuroinflammation, glial Toll-like receptor 4 (TLR4), oxidative stress, and AD. The astroglial and microglial TLR4 interact with Aβ, triggering MyD88→TRAF6→MAPK to NFκB, AP1 and inflammatory signals. Further, increased NOS and reactive oxygen species (ROS) levels induce oxidative stress and neurodegeneration.

Neuroinflammation is one of the key factors promoting AD pathogenesis, and astrocytes and microglia have a significant contributory role in it. Aβ binds to microglial and astrocytic toll-like receptor 4 (TLR4), initiating MyD88 signaling cascade, which induces the tumor necrosis factor receptor-associated factor 6 (TRAF6) effector component (Okun et al., 2009; Shi et al., 2016; He et al., 2020c). It has also been reported that knockdown or silencing of LRP1 in WT, as well as APP/PS1 double-transgenic mice, resulted in the increased nuclear NF-κB p65 subunit expression, along with enhanced TLR4, MyD88, TRAF6, neuroinflammation, microgliosis and astrogliosis in the hippocampus and cerebral cortex (He et al., 2020b, c). Another microglial membrane receptor, CD33, undergoes an increased expression in the AD brain, where its interaction with the sialic acid residues of Aβ leads to a decreased Aβ clearance. Hence, CD33 knockout in the APP/PS1 mice attenuated Aβ plaque deposition and cognitive decline, respectively (Griciuc et al., 2013; Perea et al., 2020). Moreover, the type I transmembrane glycoprotein, TREM, expressed in the microglial cells, directly interacts with several forms of Aβ, with the strongest affinity for soluble Aβ42 oligomers (Lessard et al., 2018). The elevated TREM2 expression in the 5xFAD mouse model triggered a decrease in the amyloid burden and neurobehavioral deficits (Lee et al., 2018). On the other hand, microglia-mediated phagocytosis of Tau was found to have developed through its interaction with the microglial receptor, CX3CR1, where Tau competed with the neuronal ligand CX3CL1. Corroborating this, CX3CR1 deficient mice and microglial cells demonstrated an aberrant uptake and degradation of Tau (Bolos et al., 2017). Studies in Tg2576 transgenic mice models of AD, macrophage inflammatory protein-1α (MIP-1α^−/−^), and CC-chemokine receptor 5 (CCR5^−/−^)-deficient mice demonstrated that a subsequent NF-κB and mitogen-activated protein kinase (MAPK) pathways trigger a shift from the anti-inflammatory to pro-inflammatory phenotypes in astrocytes and microglia. This promoted the generation of cytokines, like interleukin (IL6, IL1α, and IL1β), tumor necrosis factor α (TNFα), granulocyte-macrophage colony-stimulating factor, and macrophage inflammatory proteins, which stimulated AD pathogenesis (Manczak et al., 2009; Passos et al., 2009; Kaur et al., 2019). These cytokines further induce Nitric oxide synthase activity in the glial cells, enhancing NO production, lipid peroxidation, and ultimate neuronal damage (Asiimwe et al., 2016; Figure 3). Astrocytes are one of the most well-studied cell types for neuroinflammation and AD, and the activation of its reactive phenotype involves an upregulated glial fibrillary acidic protein (GFAP) expression. It has also been seen that of the different GFAP isoforms, GFAPα and GFAPδ-immunoreactive astrocytes are usually in abundance near Aβ plaques (Kamphuis et al., 2014).

Microglia, that comprised the macrophage-like resident immune cells of the CNS, enter the brain during embryonic development and further participate in neuroinflammation, as well as AD pathogenesis in the elderly (Yin et al., 2017). Microglia induce an immune response to Aβ and migrate towards the dense-core senile plaques, with increased immunoreactivity for the reactive microglial markers, CD11b, CD68, complement receptor 3, and CD45 (Mandrekar-Colucci and Landreth, 2010). A distinct association has been reported between the different Aβ forms (monomers, oligomers, and fibril and microglia) and neuroinflammation through the microglial secretion of proinflammatory cytokines (Wang et al., 2015). The microglia-induced neuroinflammation and subsequent AD pathology also relate to the participation of glutamate-associated extrasynaptic NMDA receptor and GluR1 activation and an ultimate Aβ generation and neuronal cell death, studied in the hippocampal slices of mice (Wang et al., 2004; Heneka et al., 2013). Specifically, Nlrp3(−/−) or Casp1(−/−) mutations in WT and APP/PS1 mice demonstrated reduced hippocampal and cortical inflammation, apoptosis, and Aβ deposition, as well as decreased cognitive decline (Heneka et al., 2013). Moreover, an Aβ-associated microglial NLRP3 inflammasome induced the caspase-1-dependent cleavage of pro-IL-1*β*/pro-IL-18 into IL-1*β*/IL-18, as well as lysosomal damage and cathepsin B release, which further initiated pro-inflammatory and chemotactic mediators, inflammation cascade, and neurodegeneration (Hanslik and Ulland, 2020). Increased generation of Apolipoprotein E (APOE) associates with glia-derived cholesterol dyshomeostasis (Mauch et al., 2001), altering the communication between endoplasmic reticulum and mitochondria at the mitochondria-associated ER membranes. This impairs normal mitochondrial functioning and enhances a pro-inflammatory state in the brain, as also observed in an astrocyte-conditioned media model (Tambini et al., 2016; Tzioras et al., 2019). APOE affects inflammatory environments by controlling the expression of glial genes related to immune functions, which influence the central amyloid metabolism and the etiology and pathophysiology of AD (Liu et al., 2013). Moreover, Nuclear factor erythroid 2- (NF-E2-) related factor 2 (Nrf2), which acts through the Nrf2/antioxidant responsive element pathway, is a key neuroprotective factor in AD, having a distinct link with reactive astrogliosis and microglial reactivity in the hippocampus. Corroborating this, it was observed that inflammatory microglia infiltrated and surrounded the Aβ plaque following Nrf2 ablation in APP/PS1 transgenic mice, exacerbating the generation of pro-inflammatory cytokines and neurological impairments (Ren et al., 2020). Conversely, NRF2 activator, methysticin, attenuated the hippocampal microglial reactivity, astrogliosis, neuroinflammation, oxidative damage, and long–term memory deficit in the APP/PS1 mice (Fragoulis et al., 2017).

Although the glial cells are key participants in neuroinflammation, the peripheral immune cells also infiltrate the brain and regulate AD pathology. In normal conditions, peripheral immune cells are present in the brain parenchyma. Conversely, a range of peripheral immune cells, such as macrophages, neutrophils, and lymphocytes infiltrate the brain in AD (Unger et al., 2020; Wyatt-Johnson and Brutkiewicz, 2020). Notably, the peripherally derived immune cells, such as monocytes, migrate (into the CNS) close to the Aβ deposits in AD, and further form macrophages to promote clearance of Aβ (Hohsfield and Humpel, 2015). A transgenic mouse model of AD showed that this monocyte trafficking was markedly controlled by the chemokine receptor, CCR2, which underwent a decrease in AD, enhancing cerebral Aβ deposition and cognitive decline (Naert and Rivest, 2011). Moreover, the infiltrating neutrophils served as a key source of inflammatory mediators, myeloperoxidase, ROS, and intravascular neutrophil extracellular traps (Zenaro et al., 2015; Rossi et al., 2020). The neutrophil migration into the CNS also induced damage to the vascular permeability and BBB integrity (Rossi et al., 2021). Additionally, the enhanced Aβ levels induced peripheral immune cell exhaustion, with the participation of regulatory T cells (Tregs) in amyloid clearance as well as amyloid pathology. While, the depletion of Tregs inhibited the microglial recruitment of Aβ towards amyloid deposits, selective amplification of Tregs increased the plaque-associated microglial count and spatial learning and memory loss in the transgenic APP/PS1 mice (Dansokho et al., 2016). Furthermore, the CD3^+^/CD4^+^ T-helper and CD3^+^/CD8^+^ cytotoxic T-cells were found in the brain of transgenic mice for AD (Ferretti et al., 2016; Merlini et al., 2018; Unger et al., 2018a, b). It has been reported that CD3^+^ T-cells are associated with Tau rather than Aβ amyloid plaque pathology (Merlini et al., 2018). A strong association between CD8^+^ T-cells in the brain parenchyma with microglia, neurons, and neuroinflammation has also been observed. The CD8^+^ T-cell population was regulated by microglia, with the increase in the CD8^+^ T-cells migration into the brain in microglia-depleted APP-PS1 transgenic mouse brain (Unger et al., 2018b). The removal of CD8^+^ T-cells triggered a significant change in neuronal and synaptic markers, indicating their potential participation in the AD-induced changes in neuronal functioning and synaptic plasticity (Unger et al., 2020). Moreover, Interferon gamma generated from the infiltrating Th1 cells increased plaque burden and AD pathology in the APP/PS1 mice model of AD (Browne et al., 2013).

### Glutamate Signaling

**Figure 4:**
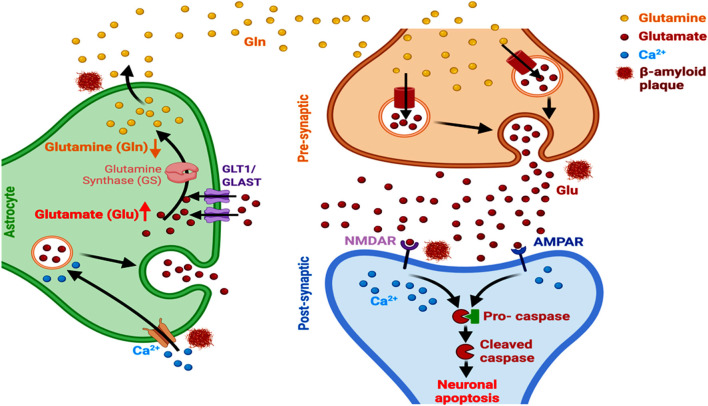
Astrocyte-dependent glutamate signaling and AD. Aβ alters the expression of GLT1 and GLAST transporters, alters glutamate-glutamine ratio, and induces astrocytic glutamate excitotoxicity and Ca^2+^ entry into the cells. This ultimately leads to a modulated activation and functioning of NMDARs and AMPAR, culminating in neuronal apoptosis in AD.

Amyloid fibrils stimulate the activity of metabotropic glutamate receptor 1 (mGluRl) that plays a key role in the regulation of synaptic plasticity and cognition, as observed in the APPsw/PSEN1DE9 mice (Bie et al., 2019; Li and Selkoe, 2020). Astrocytes express glutamate transporters, participating in the active uptake of glutamate at the synaptic and extrasynaptic sites of the CNS (Rose et al., 2017). The astrocytic glutamate-aspartate transporter (GLAST) and glutamate transporter-1 (GLT-1) reduce glutamate burden in the synaptic cleft and promote the synthesis of glutamine through increased glutamine synthase activity. Hence, a reduction in these astrocytic transporter levels accelerates the development of cognitive deficits in AD, as observed using a D-[3H]-aspartic acid uptake assay in cultured hippocampal astrocytes and neurons (Tong et al., 2017; Pajarillo et al., 2019). It has been reported that Aβ_1–42_ induces abnormal GLT-1 localization and internalization in astrocytes, inhibiting the clearance of synaptically released extracellular glutamate levels. This involves the participation of oxidative stress and inflammation pathways. Aβ_1–42_ also causes inflammation-related transcriptional alteration in GLT-1 and GLAST levels, predominantly in astrocytes, as observed in astrocytes from acute mouse hippocampal slices (Scimemi et al., 2013). A subsequent spread of glutamate, from one synaptic domain to another, enhances extrasynaptic NMDAR-mediated excitotoxicity, which restricts the normal neuronal survival, activity and networking and consequent synaptic transmission (Figure 4; Findley et al., 2019). An accompanying immediate neuronal Ca^2+^ influx, involving the activation of GluN2B-containing NMDARs, inhibits hippocampal long-term potentiation (Liu et al., 2019). Other than dyshomeostasis in extracellular glutamate level, a marked link between altered astrocytic GLT1 and complement proteins that participate in microglial synaptic remodeling has also been reported in AD. Aging involves an upregulated synaptic classical complement cascade in the CNS, initiated by C1q protein (observed in the aged C1q-deficient mice; Stephan et al., 2013) and microglia mediates the synaptic elimination through phagocytosis (Luchena et al., 2018; Lee and Chung, 2019). Additionally, GLT1 induces presynaptic metabotropic glutamate receptor activity and reduces the constitutive glutamatergic strength, amplitude and frequency of spontaneous miniature excitatory synaptic current in hippocampal CA1 neurons. The astrocyte-microglia interaction through complement activation here plays a vital role in amyloid pathology as reported in primary glial cells and hippocampal and cortical regions of APP transgenic mice (Lian et al., 2016). Moreover, the complement factors C3 and C1q that regulate innate immunity, microglia-mediated synaptic pruning, and neuronal survival undergo significant activation in the brain and promote AD pathogenesis (Hansen et al., 2018).

### Lipoprotein Dysfunction

APP, PSEN1 or PSEN2 trigger familial AD, together with cholesterol and lipoprotein dysfunction, altered peripheral lipid metabolism and polymorphic gene expression (Dai et al., 2018; Huentelman et al., 2019). LRP1, that has a significant contribution in vascular dysfunctions (Ramanathan et al., 2015), modulates Aβ generation *via* altered regulation of proteases, growth factors and an eventual inflammation (Ashok et al., 2016; He et al., 2020b). LRP1 is abundant in brain cells, and a neuronal deletion of LRP1 stimulates astrogliosis and microglial reactivity and the NF-κB and MAPK signaling pathways. Corroborating this, a reduced hippocampal LRP1 expression has been observed in the transgenic AD mice (He et al., 2020b, c).

**Figure 5:**
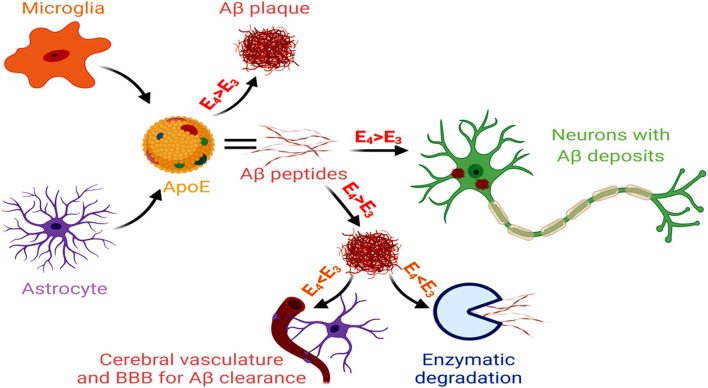
Glial cells, APOE and AD. Microglial and astrocytic APOE, with greater involvement of APOE4 > ApoE3, act as risk factors for Aβ generation and also participate in increased vascular damage. On the other hand, APOE3 > ApoE4 promotes Aβ clearance and degradation. Hence, the dysregulated APOE3 and APOE4 levels promote AD pathology.

Unlike early-onset AD, that involves genetic mutations in APP, PSEN1 and PSEN2, late-onset AD (LOAD) that comprises 95% of AD cases, shows a distinct association with the isoforms of APOE gene (Corder et al., 1993). APOE-ε4 carrier in the brain is the key genetic risk factor for LOAD, and each APO-E-ε4 allele reduces the age of AD onset (Liu et al., 2013). The ApoE isoform shows a significant contribution in AD pathogenesis, characterized by increased gliosis, Aβ accumulation and tau hyperphosphorylation. As reported in the human subjects, this ε4 variant of APOE is predominantly secreted by the astroglial cells within the brain and associates with innate immune responses (Gale et al., 2014). Inducible expression of human ApoE4 in astrocytes of mouse models at the initial phase of amyloid development results in significant Aβ aggregation and fibrillar plaque formation in the cortex and hippocampus, together with neuritic dystrophy (Liu et al., 2017). The ApoE4 isoform reduces lipoprotein-mediated cholesterol transport from astrocytes to neurons, disrupting cholesterol homeostasis in neurons and glia. It leads to an aberrant Aβ accumulation, microglial inflammatory responses, altered synapse pruning activity in astrocytes, synaptic degeneration and neuronal cell death. APOE4-containing lipoproteins reduce the uptake of oligomeric and fibrillar Aβ in astrocytes and decrease microglial Aβ clearance, resulting in increased Aβ accumulation (Lin et al., 2018). A mouse model of tauopathy showed that the ApoE4 variant exacerbated tau pathology, independent of Aβ, involving microglial reactivity and upregulation of pro-inflammatory genes (Shi et al., 2017). The altered astrocytic APOE4 gene expression often appears as the reason for Aβ and tau-induced neural circuit hyperactivity in AD. An APOE4-mediated dysregulation of the membrane-lipid composition and endolysosomal homeostasis, in association with altered intracellular cholesterol distribution, Ca^2+^ signaling, and excitability, have been observed in the astrocytes of hippocampal slices from *APOE3* and *APOE4* gene-targeted replacement mice (Larramona-Arcas et al., 2020). The APOE allele also contributes to altered gliotransmission and neural-circuit stimulation in LOAD. This involves lysosome dysregulation-mediated Ca^2+^ excitability, changes in the membrane lipidome and distribution of intracellular cholesterol, and altered Ca^2+^ responses. An astrocytic APOE-cholesterol complex undergoes transport by ATP-binding cassette transporter ABCA1 and is then absorbed into neurons through LRP1 and metabolized to 24-hydroxycholesterol by Cytochrome P450 Family 46 Subfamily A Member 1 (CYP46A1; Czuba et al., 2017). This elevated 24-hydroxycholesterol level (essentially suppresses the expression of the transcription factors SREBP-2 and SREBP-1C, LDLR and HMG CoA-reductase, which restrict cholesterol levels in astrocytes), secreted from the brain into the plasma and CSF, marks the early-onset stages of AD (Loera-Valencia et al., 2019). The astrocyte and microglia-derived APOE4 stimulates the formation and deposition of Aβ in the neurons. Additionally, APOE4 obtained from the pericytes and other cells of the NVU induce BBB damage and dysfunction, as observed in the post-mortem paraffin-embedded human frontal cortex tissue samples acquired from the University of Southern California AD Research Center (Halliday et al., 2016). Of the different APOE isoforms, the AD pathogenicity follows the sequence of apoE4 > apoE3 > apoE2. Conversely, capacity for Aβ clearance and enzymatic degradation of Aβ appears reduced for apoE4 compared to the other isoforms (Figure 5).

A direct association between LRP1 and Tau has also been reported, where their potential interaction *via* the microtubule binding repeat region of tau regulated tau endocytosis in neurons. Moreover, LRP1 deficiency in neuroblastoma cell line and mice led to reduced LRP1 interaction, resulting in decreased neuronal propagation of tau (Rauch et al., 2020). Thus, LRP1 functions as a common factor which regulates the Aβ and tau pathology in AD.

### Glia Cells (Astrocytes and Microglia), PRR, TREM2, Complement Activation, and Aβ Degradation

*Via* cell-surface pattern recognition receptors (PRRs) that sense immune stimulation in the brain, microglial cells recognize the neurodegeneration-associated molecular patterns (NAMPs) in AD (Deczkowska et al., 2018). This triggers microglial transformation from an inactive “ramified” to an activated “hypertrophic or ameboid morphology.” A microglial deficit in clearing Aβ induces characteristics of LOAD, and a genome-wide association study in AD patients showed a distinct connection between reduced chances of AD and mutations in the microglial PRRs (Hickman et al., 2018). Microglial cells express the Triggering receptor expressed on myeloid cells 2 (TREM2) along with its adaptor protein and signal transduction partner, DNAX-activating protein of 12 kDa (DAP12), which control microglial reactivity and affect neuronal fate (Konishi and Kiyama, 2018). Under physiological conditions, a tyrosine kinase-mediated phosphorylation of DAP12 regulates cytoskeletal reorganization and cytokine release (Painter et al., 2015). Here, a normal TREM2/DAP12-interaction sustains the mammalian target of the rapamycin (mTOR) pathway. This is essential for the microglial biosynthetic metabolism, while an altered TREM2 expression deregulates the morphological and functional responses of microglial autophagy and its response to Aβ, as detected using the 5xFAD mouse model and AD patients, as well as in bone marrow-derived macrophages and microglia (Ulland et al., 2017). TREM2 binds to lipids, phospholipids, glycolipids (released from damaged neurons), as well as APOE and Aβ in the extracellular milieu of the AD brain (Mecca et al., 2018). It induces microglial reactivity, pro-inflammatory cytokine release, and the vicious AD pathogenicity, as studied in a series of AD patients, showing Aβ deposition in the leptomeningeal blood vessels, hippocampal neuritic plaques, and cortical NFTs. The findings resulted from a genome, exome, and Sanger sequencing followed by a meta-analysis-based study that analyzed the TREM2 genetic variability in AD (Guerreiro et al., 2013). The TREM2 and APOE interaction also induces a phenotypic switch from the homeostatic state to a “neurodegenerative” disease-associated one reported in the APP-PS1 mice (Krasemann et al., 2017). Moreover, reduced expression of the anti-amyloidogenic ADAMs inhibits the ADAM-mediated shedding of TREM2 ectodomain and generation of soluble TREM2 (sTREM2) into the CSF. The sTREM2 augments microglial viability through activated PI3K and NFκB pathways and helps in the physiological functions of microglia (Zhong et al., 2017). Insertion of sTREM2 in the brain of 5xFAD mice promotes Aβ uptake and degradation, and provides a neuroprotective role against amyloid pathology (Zhong et al., 2019). High-resolution confocal and *in vivo* two-photon imaging of brain slices (cortex, layer IV–VI) in the AD-like transgenic 5xFAD and CRND8 mice models showed that microglial cells generate a dynamic regulation of Aβ plaque morphology from less-compacted and fibrillar plaques to more compacted neurotoxic ones (Condello et al., 2015). This involves an altered TREM2 expression and deregulated microglia-mediated sustenance of the plaque morphology, reducing Aβ clearance and neuronal dystrophy (Condello et al., 2018; Cianciulli et al., 2020).

Receptors such as, sialic acid-binding immunoglobulin-like lectin (Siglec) or CD33 bind to glycoproteins and glycolipids within Aβ plaques and auto-trigger their signaling pathways, promoting AD-induced neuronal loss (Puigdellivol et al., 2020). The CD33 and CD22 activation also inhibit the functioning of immunoreceptor tyrosine-based activation motifs (ITAM) that participate in microglial phagocytosis, reducing Aβ clearance (Zhao, 2019). The enhanced microglial expression of C-Type lectin domain containing 7A (CLEC7A), that also functions through ITAMs, promotes AD pathology *via* reduced phagocytosis, as observed in TgCRND8 mice (Hansen et al., 2018; Rothman et al., 2018). Another PRR, RAGE, binds to Aβ, and an increased microglial expression of RAGE stimulates a MAPK (P38 and ERK1/2)-mediated microglial migration, viability, and growth, inducing activation of NF-kB pathway and upregulation of IL-1β and TNFα. Aβ accentuates RAGE expression through a positive feedback loop, which attenuates hippocampal expression of acetylcholinesterase-positive neurites and axonal activity, together with increased neuronal stress and cognitive deficit, as observed in the transgenic mice with the neuronal human mutant APP and microglial RAGE expression (Fang et al., 2010). An age-induced stimulation of the microglial receptors, CR3 and C1qR, that predominantly participate in synaptic pruning during brain development, often involves their increased binding with Aβ, causing hippocampal synaptic degeneration and learning memory loss (Hong et al., 2016). Conversely, CR3 deficiency enhances proteolytic degradation of Aβ, decreases neuronal loss and increases cognitive functioning, particularly in AD pathology (Czirr et al., 2017). Thus, unrestricted activation of the complement signaling induces synaptic degeneration and memory impairment, which may be inhibited upon blocking this pathway.

The degradation of monomeric, oligomeric, and fibrillar forms of Aβ is mediated by neprilysin (NEP), insulin-degrading enzyme (IDE), and ACE, which play a defensive role (Ries and Sastre, 2016). NEP deficiency involves an increased amyloidogenicity, with augmented neuronal and peripheral inflammation, and a normal or upregulated astrocyte and microglial NEP stimulate plaque degradation and clearance (Nalivaeva et al., 2012). Microglial cells induce an incomplete degradation of Aβ involving a decreased hydrolytic activity of one or more enzymes at the microtubule organizing center and lysosomes (Sole-Domenech et al., 2016). A significant link has also been seen between astroglial APOE and Aβ-degrading enzyme in a genotype-dependent manner, resulting in abnormal amyloid metabolism and altered immune function of glial cells, neuroinflammation and AD pathogenesis (Fernandez et al., 2019). The scavenger receptors, TLRs, induce changes in NEP expression, with TLR2, TLR3, and TLR9-dependent decrease in NEP and a TLR4-mediated and APOE genotype-dependent differential modulation of NEP expression. This has been studied in mice “humanized” for APOE, termed as targeted replacement (TR) mice and the primary glial cultures isolated from the TR APOE ε3 and TR APOE ε4 mice (Graykowski et al., 2020). In fact, the APOE genotype-independent reduction in NEP has been attributed to decreased Aβ clearance (Kanekiyo et al., 2014).

A receptor-mediated endocytosis and fluid-phase pinocytosis also participate in microglial Aβ degradation, as found in the immortalized BV-2 murine microglial cell line as well as Cx3cr1/Gfp++ mice (Mandrekar et al., 2009). Purinergic G protein-coupled receptors (P2Y2 and P2Y6) take part in microglial Aβ clearance, with greater efficiency in the internalization of Aβ protofibrils compared to monomers through phagocytosis and pinocytosis (Sole-Domenech et al., 2016). The microglial degradation of Aβ monomers also occurs *via* secreted IDE without involving lysosomal degradation (Qiu et al., 1997; Vepsalainen et al., 2008). This involves the participation of clathrin- and caveolae-dependent processes through ERK and p38 kinase/JNK pathways, ultimately stimulating endocytosis or endocytic biogenesis, as reported in the study using the HMO6 human microglial cell line (Jang et al., 2015). In addition, lysosomal biogenesis, pH, and hydrolase properties of lysosomes influence astrocyte-mediated fibrillar and oligomeric Aβ clearance, as reported in a study involving primary astrocytes, immortalized mouse neural progenitor cell line, C17.2 cells, and APP/PS1 transgenic mice (Xiao et al., 2014). Moreover, the sirtuin family members that regulate energy metabolism and senescence, mainly in relation to vascular pathology and atherogenesis, facilitate the degradation of oligomeric Aβ and Tau in the primary astrocytes *via* the lysosomal pathway (Li et al., 2018).

### Genetic Factors

The rapid advancement of genome analysis technology has led to the recognition of risk factors for AD. CR1 is a complement receptor found on macrophages and microglia, and results in blood samples from AD and nondemented elderly subjects (from the National Institute on Aging Alzheimer’s Disease Centre, the Arizona AD Research Centre) suggested its strong link with AD, owing mainly to erythrocyte CR1-based mechanisms supporting pathogen clearance (Johansson et al., 2018). Granulin (GRN) is the precursor of the growth factor progranulin, expressed in neurons and microglia. GRN polymorphisms and deficiency induce aberration in lysosomal functioning, together with the enhanced and increased generation of complement factors. Increased C1q and C3 stimulate phagocytosis and alter microglial reactivity, which causes synaptic and cognitive loss in AD (Mendsaikhan et al., 2019). Polymorphism in the Interleukin (IL)-1 receptor accessory protein (IL1RAP) gene (rs12053868G), with functional receptor for IL-1α and IL-1β, stimulates microglial dysfunction. This results in reduced phagocytosis and clearance of Aβ, and increased plaque deposition in AD patients and the carriers of IL1RAP gene polymorphism (Hansen et al., 2018). Other genetic factors, such as the ATP-binding cassette transporter A7 (ABCA7; with a protective role in AD) has several variants, i.e., rs3764650, rs3752246, and rs115550680, and loss-of-function and nonsense mutations in the gene augment AD pathology. Pathogenic mechanisms that are induced during ABCA7 dysregulation also impact the properties and functioning of neurons and microglia (Aikawa et al., 2018). Other closely linked ABCA1 and ABCG1 cholesterol transporter genes (that encode the generation of high-density lipoprotein complexes) mediate the lipid-transporting activity for ApoE, indicating its participation in AD pathobiology (Koldamova et al., 2010). Additionally, ABCA1 and ABCA7 deficiencies modulate neuronal apoptosis and reduce the removal of cellular debris, promoting the accumulation of Aβ in the brain (Koldamova et al., 2010). The coding variant of phospholipase C-gamma-2 (PLCG2), expressed in microglia, regulates cytosolic Ca^2+^ influx and the inositol trisphosphate pathway, essential for suppressing AD pathomechanisms (Koldamova et al., 2010). The PI3K-regulated ABI family member 3 (*ABI3*) gene, which forms a key constituent of the Wiskott-Aldrich syndrome protein family verprolin-homologous protein regulatory complexes, controls cytoskeletal actin polymerization. Its coding variant (rs616338; p.S209F) shows a direct link with increased risk of LOAD (Kurisu and Takenawa, 2009; Sims et al., 2017). The ABI3 is expressed in the microglia of the AD brain, exhibiting a distinct association with amyloid plaques, as observed in the frontal cortex and the hippocampus of the autopsied brain from AD patients (Satoh et al., 2017). Microglial CELF1 gene variant rs1057233G, that demonstrates a link with an attenuated SP11, also reduced AD pathology through increased release of Aβ into the CSF from the brain (Hansen et al., 2018). This SPI1 encodes the transcription factor PU.1 that regulates the ABCA7, CD33, MS4A4A, MS4A6A, TREM2, TREML2 and TYROBP gene expression and controls the phagocytic activity of Aβ, seen through a genome-wide survival study in AD patients and controls from the International Genomics of Alzheimer’s Project Consortium (Huang et al., 2017). Microglial MEF2C gene, which shows a distinct connectivity with CX3C chemokine receptor 1 and tumor necrosis factor, is also a genetic risk factor for AD (Chen et al., 2016).

### Oligodendrocytes and AD

Demyelination, characterized by reduced myelin basic protein (MBP), is prominent in AD, and has been considered as a predictor for AD onset and associated neurodegeneration (Nasrabady et al., 2018). Loss in myelin integrity within the hippocampus, detected in 3xTg-AD mice models, indicates that oligodendrocytes undergo a pathophysiological assault during the disease pathogenesis (Desai et al., 2010). Monocarboxylic acid transporter 1 (MCT1) protein helps oligodendrocytes in providing metabolic support to neurons, and AD models showed a significant reduction in the brain MCT1 levels, indicative of axon damage and neuron loss (Mot et al., 2018). MRI together with advanced positron emission tomography demonstrated increased white matter damage at frontal and temporal lobes of the brain during early, preclinical, and later stages of AD, prior to any apparent clinical signs (Khan, 2018). Additionally, impairment in the recruitment, migration, proliferation, differentiation, and regeneration into oligodendrocytes of the chondroitin sulfate proteoglycan Neural/glial antigen 2 (NG2)-expressing OPCs is a common occurrence in AD. The upregulated OPC proliferation plays a protective role, where the NG2 cells surrounding Aβ plaques undergo macropinocytosis-mediated engulfment and a subsequent autophagy-lysosome-dependent internalization and degradation of Aβ (Li et al., 2013). NG2 cells are also located spatially near the microglia and astrocytes, demonstrating a close link with AD-induced neuroinflammation (Nirzhor et al., 2018).

Lipopolysaccharides (LPS), that play a key regulatory role in myelination, also undergo co-localization with amyloid plaques and perivascular amyloid in the AD brain (Zhan et al., 2018). Impaired BBB in AD facilitates the entry of LPS into the brain, where it interacts with the TLR4/CD14 receptors on the peripheral monocytes/macrophages, neutrophils, and brain microglia. The ensuing NFκB activation triggers the generation of IL-1, IL-6, and TNFα cytokines, which degrade the axon and myelin sheath and enhance AD pathogenesis (Zhan et al., 2018). Together with decreased total protein, lipid, and cholesterol levels and reduced oligodendrocyte nucleus diameter, myelin sheath forms a key lesion site in AD. It has also been observed that an abnormal myelination precedes axon defects and axon transport prior to the appearance of Aβ and tau pathology (Cai and Xiao, 2016). Supporting this, MRI scanning on a unit of cognitively asymptomatic adults enriched for AD risk revealed a marked correlation between myelin water fraction (that depicts age-related myelin alterations in the cerebral white matter), brain white matter integrity, and the Aβ and p-tau levels, predominantly at the regions affected in AD (Dean et al., 2017). A primary reason for the observation appears to be a direct LPS binding to oligodendrocytes in the white matter as well as gray matter, augmenting cytokine and free radical generation. Moreover, very high levels of cytokines trigger the death of OPC and augment mature oligodendrocyte and myelin sheath injury, causing aberrant myelin aggregation in AD (Schmitz and Chew, 2008). LPS-induced neuroinflammation also enhances cerebral APP and Aβ, which bind to TLR4, further increasing the Aβ levels *via* a positive feedback loop (Zhan et al., 2018). Here, MBP binds and degrades APP and Aβ, reducing Aβ fibrillization, which often results from LPS-induction and myelin damage, where the latter fails to prevent Aβ aggregation owing to the inhibited autolytic activity of MBP (Liao et al., 2009). The injured oligodendrocytes and myelin sheath, degraded MBP, degenerated axons, and aggregated Aβ thereby promote Aβ plaque deposition (Papuc and Rejdak, 2020).

AD involves a disordered development of oligodendrocyte, characterized by an unevenly thick myelin sheath, reduced internodal gap, and enhanced NG2 and 2’,3’-Cyclic-nucleotide 3’-phosphodiesterase in the oligodendrocyte lineage cells. This has also been detected through histological and electrophysiological studies of the hippocampus and behavioral assays in the human platelet-derived growth factor beta polypeptide platelet-derived growth factor beta polypeptide (PDGFB)-APP^Sw.Ind^ transgenic mice (Ferreira et al., 2020). Oligodendrocyte development is critically linked to neuregulin expression from axons tightly regulated by caspase-6 activation. The AD-induced altered myelin morphology shows a critical association with caspase-6-dependent neuregulin type III cleavage (Hu et al., 2016). An increased neuregulin III expression induces hypermyelination, deregulates myelin morphology, alters electrical impulse along the axon and at the nerve terminals and induces an ultimate temporal and spatial progression of cognitive impairment (Hu et al., 2016). White matter alterations participate in AD pathogenesis, involving inflammatory responses. Presenilin and APP mutations enhance the susceptibility of oligodendrocytes towards stress and alter axon-mediated information to cells, affecting normal myelination (Nasrabady et al., 2018). It has also been hypothesized that myelin degeneration in the late-myelinating white matter tracts releases iron that enhances AD pathogenicity *via* oxidative stress (Raz and Daugherty, 2018).

Aβ, *via* an oxidative mechanism, triggers the activity of sphingomyelinase (NSMase)-ceramide in the cell membrane, releasing ceramide that has pro-apoptotic properties and a subsequent oligodendrocyte dysfunction (Jana et al., 2009). Moreover, inflammation and oxidative stress have been found to be the two key factors relating oligodendrocyte death to the NFTs (Cai and Xiao, 2016).

Clinical as well as histological studies demonstrated a distinct link between white matter degeneration and myelin atrophy in AD, where the white matter-induced neurodegeneration resulted from localized or remote cerebral injury (Nasrabady et al., 2018). A difference in the distribution pattern for white matter damage has been observed, and LOAD involved damage to the medial temporal region. Moreover, early-onset AD affected the white matter of the lateral temporal cortex, parietal cortex, cingulum, and corpus callosum (Migliaccio et al., 2012). Increased Aβ levels also demonstrated a distinct link with oligodendrocyte death, as a key feature of white matter demyelination, which further augmented neurodegeneration (Nasrabady et al., 2018).

AD features signs of leukoaraiosis in the white matter region, associated with demyelination, glial cell death, and intercellular edema in the epidermis (Rosenberg et al., 2016). The extent of leukoaraiosis appears correlated with the severity of cognitive fall and functional alteration in AD, as reported from a multicenter study comprising patients with AD and mild cognitive impairments (Sarabia-Cobo et al., 2014). WMH increases with age, hypertension and, the blood homocysteine, IL-1, and inflammation-induced C-reactive protein levels, having a distinct link with the pathobiology of AD. Moreover, APOE ε2 allele, IL-1β-511T allele homozygotes, and CRP-286T allele carriers that promote inflammation related to WMH, demonstrate a key link with AD pathology (Raz et al., 2012).

## Vascular Pathology, Brain Cells, and AD

A marked overlap exists between cerebrovascular dysfunction, loss of neurovascular integrity, and AD, with reported cases of lacunar infarcts in more than 70% of AD patients (Govindpani et al., 2019). The hypoxia-induced insufficient CBF impairs the normal endothelial activity, inducing vasoconstriction. Thus, vascular complication involves the accumulation of toxic substances from the blood, resulting in decreased glucose and oxygen levels in the brain (Morikawa et al., 2012).

The BBB, comprised of microvessels and endothelial cells (in the brain capillaries) along with pericytes, astrocytic end-feet, microglia, and neuronal processes of NVU, restricts the undesired exchange of toxic materials between the CNS and systemic blood circulation. A dysfunctional NVU and BBB may precede AD pathology, involving extracellular Aβ-associated reactive astroglia and microglia, vascular wall thickening, reduction in smooth muscle cells, and neuritic loss (Soto-Rojas et al., 2021). The subsequent induction of astrocyte and microglia-induced inflammatory mediators, such as IL-1, TNFα, and the complement component subunit 1q (C1q; Liddelow et al., 2017), further enhance NVU and BBB damage through a feedback loop, with the accelerated generation of p-tau and NFT. The NVU-linked microglial cells associate with Aβ *via* CR1 (CD35) fAβ receptors, which appears as a critical reason for altered BBB and NVU integrity (Fonseca et al., 2016; Soto-Rojas et al., 2021). Microglial participation through increased contact size at the basement membrane is a key to the development of perivascular glia limitans surrounding the brain capillary vessels of the NVU, particularly during inflammation. This induces vascular permeability, endothelial phagocytosis, and altered leukocyte extravasation, as studied in a Middle cerebral artery occlusion model and wild-type C57Bl/6 mice and CX3CR1+/GFP mice (Jolivel et al., 2015; Joost et al., 2019). The reactive microglia promote BBB dysfunction, accompanied by dysregulated reorganization and signaling of tight junction proteins and controlled transendothelial electrical resistance in AD (Sumi et al., 2010; Zenaro et al., 2017). Additionally, the reactive microglia secrete excess IL-1β, which reduces BBB integrity, promotes vascular permeability and augments neutrophil trafficking through the BBB, as observed in AD patients (Zenaro et al., 2015). The VEGF that mediates a microglial chemotactic response undergoes an upregulation in AD, attracting microglia around blood vessels and parenchyma, which co-occurs with neovascularization and decreased blood flow (Ryu et al., 2009; Zhao et al., 2018). An increased VEGF expression is also correlated with angiogenesis and inflammation following Aβ peptide insertion in the rat brain hippocampus (Zand et al., 2005).

However, BBB integrity reduces with age and brain inflammation (Daneman and Prat, 2015). Since astrocytes are one of the fundamental cellular components of the BBB, reactive astrogliosis and a loss of end-feet processes in the aging brain act as contributing factors for BBB disruption and reduced Aβ efflux (Yamazaki and Kanekiyo, 2017). Astrocytes, *via* end-feet processes, associate with thousands of synapses and capillaries for neurovascular coupling, leading to a regulated CBF and signaling in response to neuronal activity (MacVicar and Newman, 2015). Moreover, astrocytes express LRP1 that causes Aβ efflux from the brain through the BBB (Sagare et al., 2012). Hence, a cerebrovascular injury-induced astrocyte damage and altered BBB permeability cause faulty clearance of Aβ, accentuating AD pathology (Erickson and Banks, 2013; Sweeney et al., 2018). Moreover, an altered expression, distribution, polarization, and mislocalization of aquaporin-4 (AQP4), at the astrocyte endfeet surrounding blood vessels and blood CSF barrier, lead to BBB impairment. AQP4 sustains the astroglial water transport, and its altered expression and functioning hinder the normal CSF flux into the parenchyma, hamper solute clearance through interstitial fluid (ISF) drainage and disturb cerebrovascular Aβ clearance. Increased thickening of the vascular basement membrane and altered cellular and extracellular constituents occur during aging, impeding the drainage of Aβ through the ISF.

Endothelial basement membrane anchors cerebral and vascular cells at the BBB. On the other hand, astrocytes help in the formation of the parenchymal basement membrane, rich in the extracellular matrix proteins, laminin α1, and α2 isoforms, that organize the spatial arrangement and physical integrity of the gliovascular interface (Thomsen et al., 2017). In addition to the astrocytes, pericytes associate with endothelial cells and regulate the soluble platelet-derived growth factor receptor β in the CSF. Thus, a disproportionate astrocyte-pericyte-endothelial interaction and pericyte deficit stimulate cerebral Aβ deposition and CAA formation (Yamazaki and Kanekiyo, 2017).

Vascular dysfunctions are normally observed at the initial stages of AD pathogenesis, with the local cerebral hypoxia playing a contributory role (Salminen et al., 2017). *In vitro* and *in vivo* studies showed that hypoxia enhances the expression and activity of BACE 1, reduces the functioning of α-secretase proteins (ADAM10 and ADAM17) and neuroprotective sAPPα levels. Hypoxia restricts NEP activity in neurons, astrocytes, and vascular cells, leading to enhanced Aβ generation and accumulation together with glutamate excitotoxicity in the hypoxic/ischemic brain (Auerbach and Vinters, 2006; Marshall et al., 2006; Sun et al., 2006, 2012; Fisk et al., 2007; Guglielmotto et al., 2009; Grammas et al., 2011; Pluta et al., 2016). Increased levels of Hypoxia inducible factor 1-alpha (HIF-1α) in the microcirculation of AD patients play a key contributory role in aggravating angiogenesis and vascular activation (Grammas et al., 2011). HIF-1α functions as a vital regulator of immune cell responses in inflamed tissues (Mcgettrick and O’neill, 2020). HIF-1α stimulates the activation of NFκB *via* cross-talk, resulting in neuroinflammation and generation of cytokines, ROS, and NO in the brain (Lukiw et al., 2003). This could resist the functioning of the insulin and insulin like growth factors (IGF)-1 and 2, alter glucose uptake and metabolism, trigger aberrant mitochondrial (mt) activity and reduce oxidative phosphorylation, which further triggers a mtDNA damage, reduces adenosine triphosphate formation, activates glycogen synthase kinase-3β (GSK-3β)-induced tau phosphorylation and increases Aβ formation (Ashok et al., 2017). Both hypoxia and AD associate with energy metabolic deficiencies and oxidative stress, and cerebral microvessels from AD patients release thrombin, VEGF, angiopoietin-2 (Ang-2), MMPs as well as inflammatory proteins, which correspond to the hypoxic responses (Grammas and Ovase, 2001; Grammas et al., 2006). The hypoxia-induced altered blood vessel function, capillary diameter, and endothelial metabolic properties synergize with AD pathology to induce dementia. The reactive astroglia, microglia, and inflammatory macrophages surround angiogenic factors in cerebral tissues, where the Prostaglandin E2 mediates the inflammatory responses and hypoxia-associated cellular angiogenesis in AD (Ko et al., 2015). The participation of inflammation in hypoxia-induced AD pathology is also evident from the study showing an IL-1β-mediated generation of VEGF gene expression in AD (Pogue and Lukiw, 2004; Grammas, 2011). Overall, reactive glial cells are abundant near parenchymal fibrillar amyloid plaques and inflammatory mediators (Mandrekar-Colucci and Landreth, 2010). Additionally, in CAA type-1, marked by the extension of vascular amyloid angiopathy and perivascular clearance, a strong expression of reactive astroglial cells and microglia has been observed around dyshoric Aβ-laden capillaries.

## Conclusion

Damage to the vascular-neuronal axis leads to the pathogenesis of AD. The glial cells have a significant impact on AD pathogenesis, amyloid plaque formation, and cerebrovascular damage, involving diverse pathological pathways and mechanisms. The process results in dendritic loss, synaptic nerve damage, altered neurotransmission, and an overall hippocampal and cortical degeneration.

The neuronal plaques penetrate through the glial cells and macrophages, and further into the capillaries and cerebral parenchyma. This progression associates with glial scarring, which leads to increased fibrillogenesis and its infiltration into the cell membrane and cytosolic process, together with evidence of immune dysfunction and DNA damage as well. In association with this, a dysregulated functioning of neurovascular and gliovascular units, loss in BBB integrity, altered angiogenesis and neurogenesis, particularly in association with stroke, cardiovascular and cerebrovascular risk factors, cerebral and vascular injury, and neuroinflammation have been observed in AD. Hence, targeting the glial cells, immunomodulatory pathways, and NVU appears important in reducing neurodegeneration. Overall, novel therapies reducing glial dysfunction and compromised vascular functioning may have a significant role in improving neuronal integrity, and triggering increased cognitive function and reduced dementia in AD.

## Author Contributions

I have written, compiled, and arranged the manuscript.

## Conflict of Interest

The author declares that the research was conducted in the absence of any commercial or financial relationships that could be construed as a potential conflict of interest.
